# Supplementary material for: Risk factors for sacrococcygeal pilonidal sinus: a systematic review and meta-analysis supplemented by genetic causal assessment
Source: Front Surg. 2026 Jan 7;12:1718589. doi: 10.3389/fsurg.2025.1718589 (PMC12819706; doi:10.3389/fsurg.2025.1718589)
Supplement: Supplementary file 2 [file Datasheet2.zip › Supplementary Data 2/MR_pipeline_p5e-8/leaveone_plots/ebi-a-GCST90012105.finngen_R12_L12_PILONIDALCYST.pdf]

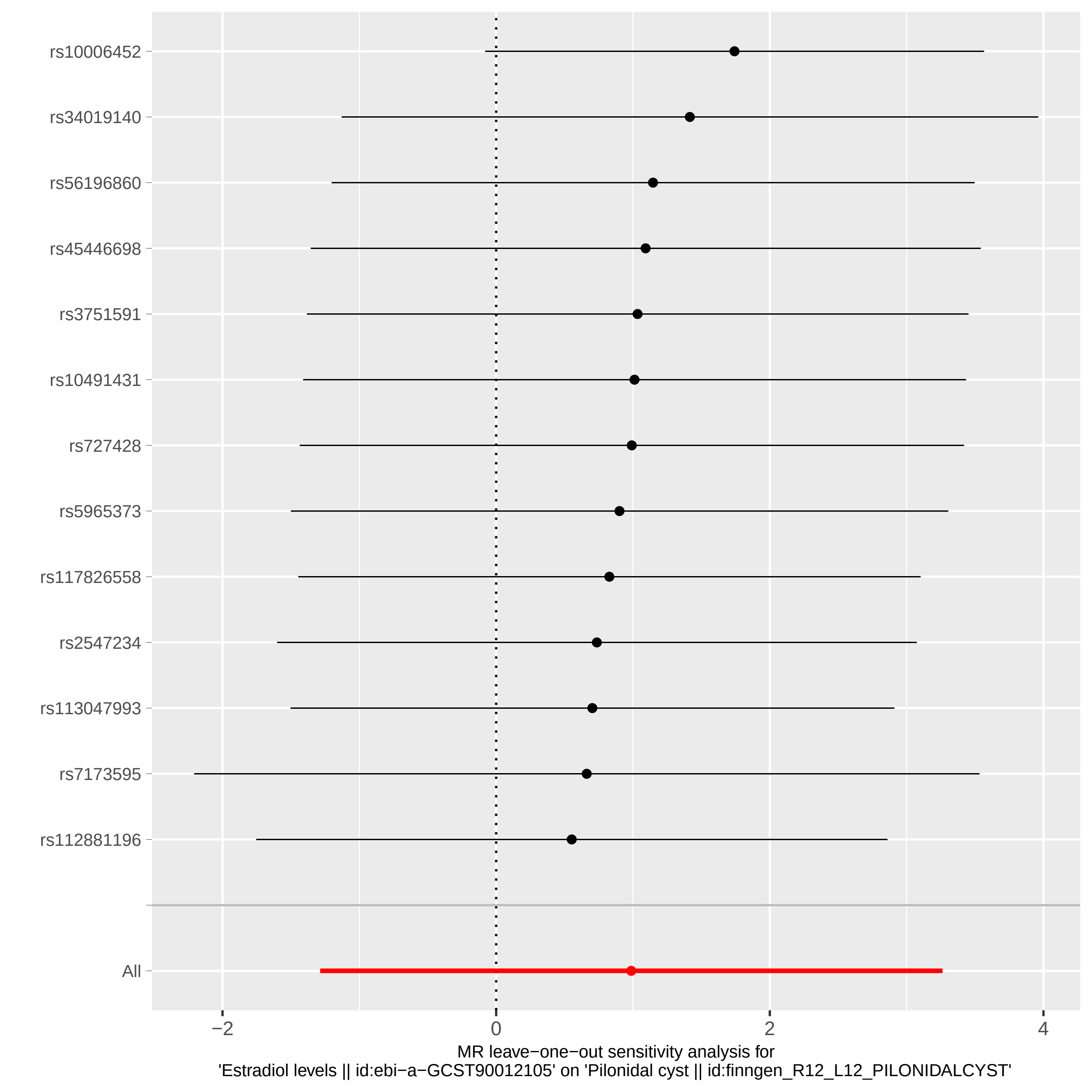
rs10006452
rs34019140
rs56196860
rs45446698
rs3751591
rs10491431
rs727428
rs5965373
rs117826558
rs2547234
rs113047993
rs7173595
rs112881196
All
−2
0
2
4
MR leave−one−out sensitivity analysis for
'Estradiol levels || id:ebi−a−GCST90012105' on 'Pilonidal cyst || id:finngen_R12_L12_PILONIDALCYST'
